# Relapsing Fever

**Published:** 1915-12

**Authors:** 


**Relapsing Fever.** 5 cases have been reported by Meader, all but one occurring in a camping party on Bear Creek, Colorado. The camp had frequently been visited by gypsies. Col. Med. October.

